# The mediating role of obesity on the prospective association between urinary sucrose and diabetes incidence in a sub-cohort of the EPIC-Norfolk

**DOI:** 10.1038/s41387-023-00243-5

**Published:** 2023-09-02

**Authors:** Alexander Lang, Oliver Kuss, Tim Filla, Gunter Kuhnle, Sabrina Schlesinger

**Affiliations:** 1 Institute for Biometrics and Epidemiology, German Diabetes Center, Leibniz Institute for Diabetes Research at Heinrich Heine University Düsseldorf, Auf’m Hennekamp 65, D-40225 Düsseldorf, Germany; 2 Centre for Health and Society, Medical Faculty, Heinrich-Heine-University Düsseldorf, Germany Institute for Biometrics and Bioinformatics, University Hospital, Düsseldorf, Germany; 3 German Center for Diabetes Research, Partner Düsseldorf, München-Neuherberg, Germany; 4 Department of Rheumatology, Medical Faculty, Heinrich Heine University Düsseldorf, Düsseldorf, Germany; 5 Department of Food & Nutritional Sciences, University of Reading, Reading, RG6 6DZ United Kingdom

**Keywords:** Risk factors, Type 2 diabetes

## Abstract

**Background/objectives:**

Findings from epidemiological studies showed controversial findings between dietary sugar intake and the development of diabetes. Most of these studies assessed dietary sugar intake by self-reports which might be prone to bias. Urinary sucrose, an objective biomarker of sucrose intake, might provide better insights into this association. Thus, the aim of this study was to investigate the associations between sucrose intake, measured via self-reports and urinary sucrose, with incident diabetes and to detect the impact of obesity on this association.

**Subjects/methods:**

Data of a sub-group (*n* = 2996) from the prospective EPIC-Norfolk cohort were investigated. Sucrose intake was assessed by self-reports (validated food frequency questionnaire (FFQ) and 7-day diet diaries (7DD)) and as an objective urinary sucrose biomarker. Cox proportional hazard models were conducted to calculate hazard ratios (HRs) and 95% confidence intervals (CI) for the associations between urinary and dietary sucrose intake and incident diabetes. Mediation analysis was performed to investigate the mediated percentage of body mass index (BMI) and waist circumference (WC) on this association.

**Results:**

The mean age of the participants was 60.6 ± 9.5 years and 53% were women. After a mean follow-up of 11.2 ± 2.9 years, 97 participants developed diabetes. Findings suggested inverse associations regarding incident diabetes for self-reported sucrose intake per 50 g/d via 7DD [HR: 0.63 (95% CI: 0.43, 0.91)], and a tendency via FFQ [HR: 0.81 (95% CI: 0.46, 1.42)]. Urinary sucrose indicated a positive association with incident diabetes for each increase of 100 µM [HR: 1.14 (95% CI: 0.95, 1.36)]. The proportion mediated of BMI and WC for this association was 16 and 22%.

**Conclusions:**

These findings indicate that sucrose measured as objective urinary biomarker points to a positive association with incident diabetes. BMI might partly mediate this association. However, to obtain more precise results, more studies are warranted that consider this objective biomarker.

## Introduction

Type 2 diabetes (T2D) is a metabolic disease, which is caused by genetic components as well as environmental risk factors. Lifestyle factors, including healthy dietary behavior, play an important role in the development and progression of T2D [1]. There is a high certainty of evidence that high consumption of sugar-sweetened beverages is associated with an increased incidence of T2D [2–4]. Moreover, several observational studies investigated the association between dietary sugar intake as a nutrient and incident T2D. However, those findings were inconsistent, showing inverse [5] or null associations [6–11] for total sugar intake with T2D, as well as inverse [12] or null associations [6–8, 11, 13, 14] for sucrose intake. A systematic review and meta-analysis that pooled these studies, indicated an overall inverse association between a high intake of sucrose and incidence of T2D, and an inverse, but imprecisely estimated, association for total sugars [15]. The certainty of the evidence of these observations was rated as low or very low [4]. However, these findings should be interpreted with caution, in particular as in observational studies the assessment of dietary sugar intake is usually relying on self-reports of the participants using different forms of questionnaires. In general, this leads to a higher risk of bias, since individuals, especially overweight and obese individuals, tend to underreport their true intake of unhealthy—“high sugar”—foods [16, 17].

In consequence, biomarkers of sugar intake were previously used instead of questionnaire data in several studies to objectively assess dietary sugar intake, e.g. urinary sucrose and fructose. In a subsample of the European Prospective Investigation into Cancer in Norfolk (EPIC-Norfolk) sucrose and fructose were measured from spot urine samples, and a positive association between urinary sucrose and the development of overweight or obesity were observed, but interestingly, inverse or null associations were observed using self-reported dietary sugar intake [18, 19]. Moreover, there is an ongoing discussion, if a high dietary sugar intake contributes to an overload of calories that may lead to overweight and obesity, which is the causal risk factor for T2D [20]. However, it remains unclear, if this relationship is only influenced via the indirect pathway via overweight and obesity, or if other direct pathophysiological mechanisms also play a role. In this context, we previously conducted a global, ecological mediation analysis, which suggested that the association between per capita sugar intake and the prevalence of diabetes is mediated by the body mass index (BMI) to a large proportion, but also other mechanisms might have an influence [21]. However, the impact of BMI or waist circumference (WC) on the relation between dietary sugar intake and diabetes incidence had never been investigated in a mediation analysis by using individual data.

Thus, the aim of the present study was to investigate the mediation effect of BMI or WC on the association between dietary sugar intake assessed via urinary sucrose biomarker, food frequency questionnaire (FFQ), or 7-day diet diary (7DD) and the incidence of diabetes using prospective data from a sub-cohort of the EPIC-Norfolk study.

## Participants and methods

### Study participants

EPIC-Norfolk is a prospective cohort study, in which 77,630 healthy individuals at age 39–79 years were invited to participate between 1993 and 1997, of whom 25,639 attended the first health examination [18]. All participants gave signed informed consent and the study received ethical approval by the Norwich District Authority Ethics Committee.

**Fig. 1:**
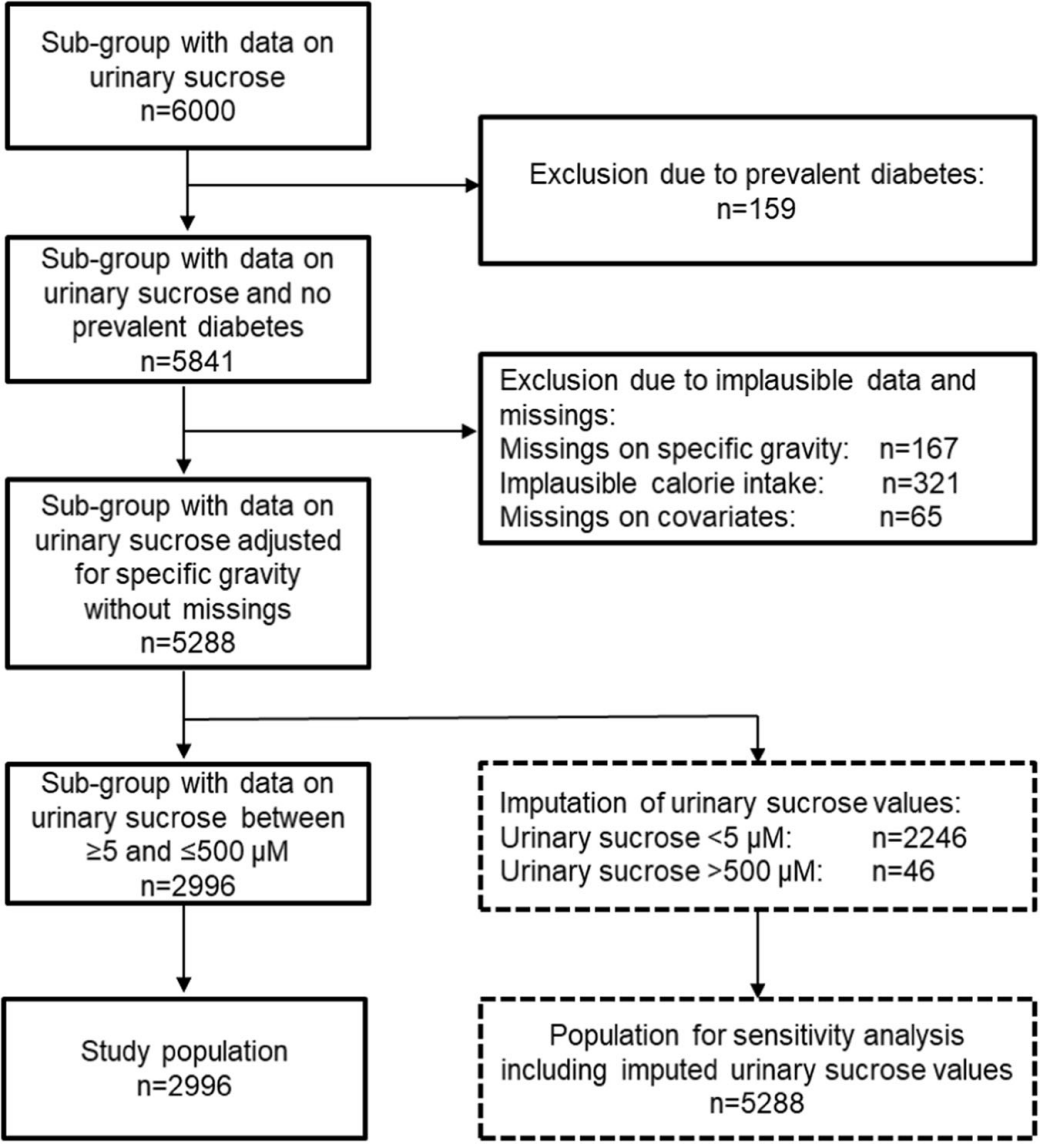
Flowchart of data preparation for final study population with urinary sucrose (*n* = 2996).

Baseline spot urine samples were available for 6 000 participants (Fig. 1). Due to prevalent diabetes at baseline, we had to exclude 159 participants. Further, participants with missing variables on specific gravity (*n* = 167) or covariates (*n* = 65), and implausible calorie intake (*n* = 321) were also excluded from our analyses. Implausible values for calorie intake were defined as <500 or >3500 kcal/day for women and <800 or >4000 kcal/day for men [22]. To adjust for the urine concentration, a quotient was generated for urinary sucrose relative to specific gravity. Participants with urinary sucrose concentrations outside the acceptable range for sucrose 5–500 µM (*n* = 2292) were excluded from our main analysis [18]. The final study population included 2996 participants without prevalent diabetes. For sensitivity analyses, multiple imputation was performed for individuals with urinary sucrose levels outside the range of 5–500 µM. The present study is reported according to the Strengthening the Reporting of Observational Studies in Epidemiology-Nutritional Epidemiology (STROBE-nut) checklist [23].

### Assessment of sucrose

Dietary sucrose intake was assessed at baseline by a 7DD and a 130-item semi-quantitative FFQ. Spot urine samples were collected at baseline health examination and analyzed for urea, creatinine, glucose, sucrose, and fructose concentration using methods previously described [18, 24]. The specific gravity of spot urine samples was measured by Multistix reagent strips (Bayer) at collection [19].

### Outcome assessment

Incident diabetes was detected by self-reports of participants from follow-up health and lifestyle questionnaires, e.g. in terms of diagnosis of diabetes by a doctor or taking any antidiabetic medication. Moreover, external sources such as the general practice register, local hospital diabetes registers, hospital admissions data with screening for any diabetes-related admissions, and Office of National Statistics mortality data with coding for diabetes were consulted to receive information on the participants’ diabetes status. To consider the diagnoses as a verified case, self-reported diabetes had to be confirmed by any of the listed external sources [25].

### Covariates

The covariates at baseline included in our analyses were selected a priori based on the literature and are illustrated with directed acyclic graphs in Supplementary Fig. 1. BMI or WC were used as mediators. Anthropometric data were measured according to a standardized protocol conducted by trained research nurses at baseline and a second health check (2HC) after three years [26]. Total energy intake was obtained at baseline by 7DD and FFQ, respectively. General factors and demographics, including education level, social class, smoking status, and family history of diabetes were assessed by questionnaires at baseline. The physical activity level at baseline, including occupational and leisure activity, was assessed by a validated questionnaire [27].

### Statistical analysis

The associations between urinary and dietary sucrose (FFQ and 7DD) with diabetes incidence were investigated by calculating hazard ratios (HR) with 95% confidence intervals (95% CI) in Cox proportional hazard models. We tested the assumption of proportional hazards (1) by the Schoenfeld residuals method for urinary and dietary sucrose (Supplementary Fig. 2), and (2) by calculating a Kaplan–Meier Plot (Supplementary Fig. 3). In the Cox model, the time of follow-up began with recruitment (assessment of sucrose) and ended with diagnosis of incident diabetes, date of death or date of last contact (31.12.2006). We investigated log-transformed urinary sucrose (per log 100 µM) and dietary sucrose (per log 50 g/d) [15] as continuous measures and calculated quartiles for intake of sucrose from FFQ, 7DD, and urine sample, respectively, by using the first quartile as reference group.

Analyses were adjusted for age and sex (model 1), and model 2 was additionally adjusted for total energy intake from FFQ or 7DD in kcal/d, education level (none, O-level, A-level, degree), smoking status (current, former, never), physical activity level (inactive, moderately inactive, moderately active and active) and family history of diabetes (yes, no). Further adjustment for social class (professional, managerial and technical, non-manual skilled, manual skilled, partly skilled, and unskilled) did not change the results remarkably (HRs changed < 5%), and thus, it was not included in the final model. In order to investigate whether obesity influences the association between sucrose and incident diabetes, additional models were adjusted for BMI or WC plus height. To test for linear trend (*p*_Trend_) the medians of the quartiles were used as continuous variables in the corresponding models. Non-linear relations between urinary sucrose and HRs of incident diabetes were explored by generating smoothing splines. After investigating several spline functions with different degrees of freedom, we chose the splines with three degrees of freedom as the best fit.

**Fig. 2:**
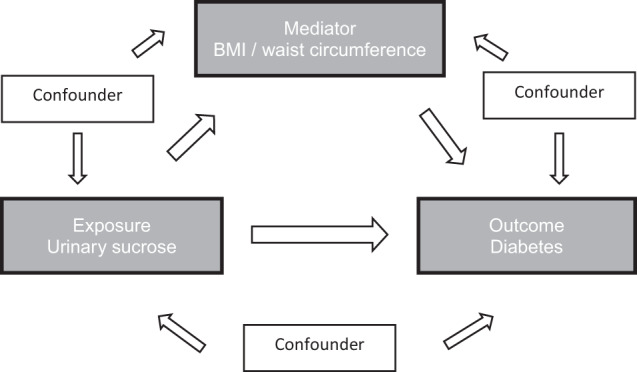
Chart of the causal pathways regarding the association between urinary sucrose and incident diabetes. The figure shows the potential mediation effect of body mass index (BMI) or waist circumference on the association between urinary sucrose and incident diabetes by considering potential confounders.

Mediation analysis was conducted according to the method suggested by VanderWeele using the mediator package in R [28, 29], investigating the indirect effect of BMI and WC on the association between urinary sucrose and the incidence of diabetes. In general, mediation analysis aims to identify the underlying mechanism of an exposure-outcome association by dividing the total effect into a direct and an indirect effect via a third, the “mediating” variable [30]. Given a counterfactual approach, the pure direct effect (PDE) is defined as the contrast in the counterfactual outcome if a person was exposed versus the same person was not exposed, assuming that the mediator remains constant at the value it would have taken if the person had not been exposed [31]. In this study, the PDE describes the effect size of the association between exposure (urinary sucrose) and outcome (diabetes incidence), assuming that the exposure does not affect the mediator (BMI or WC). In addition, the total indirect effect (TIE) is defined as the contrast in the counterfactual outcome of the value that the mediator would have taken if the person had been exposed versus the value that the mediator would have taken if the person had not been exposed, assuming that the person was fixed to being exposed [31]. Thus, the TIE represents the effect size described by the pathway of the exposure to the outcome via the mediator (Fig. 2, Supplementary Fig. 1). In addition, the proportion mediated (PM) can be calculated as the quotient of the TIE and total effect, to determine to what extent (in %) the exposure-outcome association can be explained by the influence of the mediator.

For mediation analysis, the following criteria should be fulfilled: I) the exposure (urinary sucrose intake) is associated with the outcome (diabetes incidence), II) the exposure is associated with the mediator (BMI or WC), and III) the mediator is associated with the outcome. Thus, to assess the first criterion, we conducted adjusted Cox proportional hazard models, as previously described. Further, we performed linear and logistic regression analyses to investigate the association between urinary and dietary sucrose intake and BMI or WC, respectively (criterion II). In the logistic regression models, BMI and WC were categorized as binary variables, to calculate odds ratios (OR) along with 95% CI in relation to quartiles of urinary and dietary sucrose via FFQ and 7DD, as well as log-transformed continuous urinary sucrose values. For this, individuals were divided into obese (BMI ≥ 30 kg/m^2^; WC ≥ 88 cm (f) and ≥ 102 cm (m)) and non-obese (BMI < 30 kg/m^2^; WC < 88 cm (f) and < 102 cm (m)). The third criterion was assessed by conducting Cox proportional hazard models to investigate the association between BMI or WC (continuously) and diabetes incidence.

In order to incorporate participants with urinary sucrose levels outside the detection limit in sensitivity analyses, we predicted sucrose values outside the detection range using all covariates included in the adjusted model (model 2). Participants were ordered by their predicted value and allocated to equidistant values from 0.0 to 5.0 µM for measured urinary sucrose below the detection limit and from 500 to 1000 µM for measured urinary sucrose above the detection limit, respectively. Urinary sucrose was then categorized into five groups. For the first category of urinary sucrose, the imputed values of all participants below the detection limit of 5 µM were summarized in one group (n = 2246). The remaining participants (*n* = 3042) with values in and above the detection limit were divided into quartiles. Moreover, in further sensitivity analyses, data on BMI and WC from 2HC after three years was included in regression and mediation analyses (*n* = 1728), in order to consider the time sequence of the causal pathway between sucrose intake and overweight and obesity for mediation analysis. In addition, the causal effects of the mediation analysis were estimated as a function of time to examine whether temporal variations in the hazard ratios (Q4 vs. Q1) occurred over the follow-up period [32]. All statistical analyses were performed in R 4.1.0.

## Results

In this analysis, 2996 participants (mean (SD) age: 60.6 ± 9.5 years, 52.5% women) with measured urinary sucrose levels within the detection limit were included. During a mean follow-up time of 11.2 ± 2.9 years, 97 cases of incident diabetes occurred. Men tended to have higher urinary sucrose values compared to women (Table 1). In the highest quartile, participants were more likely to be older, obese, and inactive, and to have a lower education and higher energy intake compared to participants in the lower quartiles. For sensitivity analyses, 5288 participants (mean age: 60.1 ± 9.3 years, 57.4% women) were included after imputation of sucrose values outside the detection ranges, of whom 165 developed incident diabetes (Supplementary Table 1).

**Table 1:** Baseline characteristics of participants in a sub-group of the EPIC-Norfolk divided into quartiles of urinary sucrose (*n* = 2996).

	Q1 (5.0–13.7 µM)	Q2 (>13.7–32.6 µM)	Q3 (>32.6–73.0 µM)	Q4 (>73.0–499.9 µM)	Total (5.0–499.9 µM)
*n*	749	749	749	749	2996
*n* incident diabetes	21	23	24	29	97
Age [years]	59.5 ± 9.3	60.3 ± 9.4	60.2 ± 9.4	62.3 ± 9.5	60.6 ± 9.5
Sex [m/f]	38.9%/61.1%	47.4%/52.6%	48.2%/51.8%	55.7%/44.3%	47.5%/52.5%
Body mass index [kg/m²]	26.0 ± 3.8	26.1 ± 3.7	26.4 ± 3.8	26.6 ± 3.9	26.3 ± 3.8
<25 kg/m²	44.2%	40.1%	38.1%	38.3%	40.2%
25–<30 kg/m²	43.7%	46.6%	46.2%	43.8%	45.1%
≥30 kg/m²	12.1%	13.4%	15.8%	17.9%	14.8%
Waist circumference [cm]	86.6 ± 12.2	87.8 ± 12.0	88.9 ± 11.7	90.5 ± 12.5	88.5 ± 12.2
<80 cm (f), <94 cm (m)	49.4%	46.2%	44.7%	43.3%	45.9%
80– < 88 cm (f), 94– < 102 cm (m)	29.1%	29.8%	30.0%	28.3%	29.3%
≥88 cm (f), ≥102 cm (m)	21.5%	24.0%	25.2%	28.4%	24.8%
Height [cm]	166 ± 9.0	167 ± 9.0	167 ± 9.4	168 ± 9.4	167 ± 9.2
Total energy (FFQ) [kcal/day]	2012 ± 524	2031 ± 550	2082 ± 588	2132 ± 598	2064 ± 567
Total energy (7DD) [kcal/day]	1923 ± 481	1951 ± 513	1982 ± 535	2003 ± 541	1965 ± 519
Education level					
None	37.1%	37.0%	39.3%	45.5%	39.7%
O-level	10.5%	10.8%	9.6%	9.2%	10.0%
A-level	39.7%	38.3%	38.5%	36.4%	38.2%
Degree	12.7%	13.9%	12.7%	8.8%	12.0%
Smoking status					
Current	8.1%	6.8%	9.5%	9.2%	8.4%
Former	42.9%	46.5%	44.9%	49.3%	45.9%
Never	49.0%	46.7%	45.7%	41.5%	45.7%
Physical activity level					
Inactive	26.6%	29.1%	27.6%	33.1%	29.1%
Moderately inactive	30.4%	30.0%	28.2%	27.5%	29.0%
Moderately active	23.5%	23.9%	24.0%	21.2%	23.2%
Active	19.5%	17.0%	20.2%	18.2%	18.7%
Family history of diabetes					
Yes	13.6%	10.3%	11.6%	11.9%	11.8%
no	86.4%	89.7%	88.4%	88.1%	88.2%

**Fig. 3:**
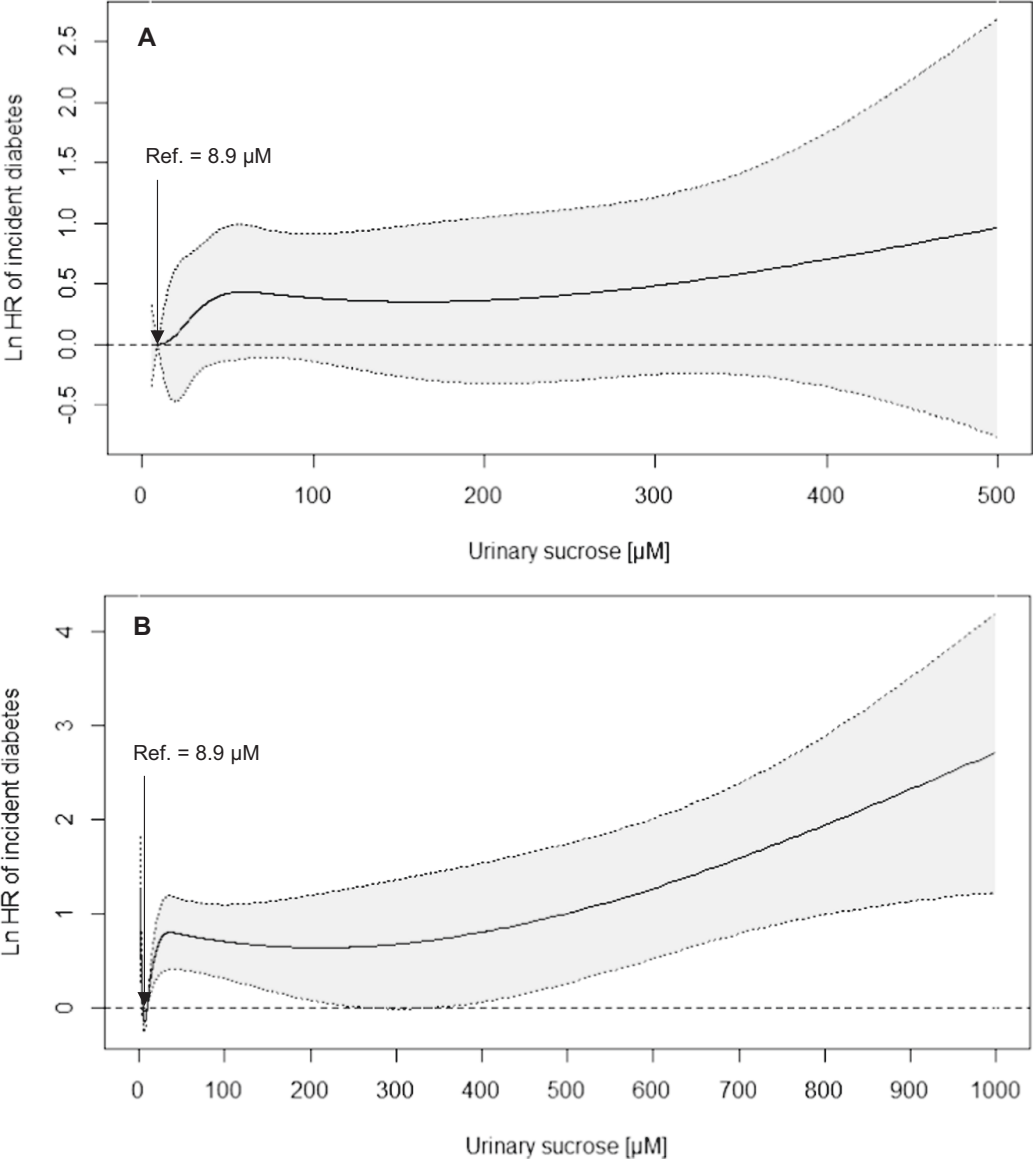
Dose–response association between urinary sucrose and incident diabetes. Smoothing splines of the association between urinary sucrose and incident diabetes before imputation (**A**—*n* = 2996) and after imputation of values of urinary sucrose levels outside the detection limit (**B**—*n* = 5228).

After the 11.2-year follow-up, results of the Cox proportional hazard models (model 2) pointed to inverse associations between dietary sucrose intake via FFQ and 7DD with diabetes incidence, and to a positive association for urinary sucrose (Table 2). Modeling sucrose as a continuous variable showed a 37% lower incidence of diabetes per 50 g/d for sucrose assessed with 7DD [HR (95% CI): 0.63 (0.43, 0.91)] and a 19%, but imprecisely estimated, lower incidence of diabetes for sucrose assessed with FFQ [HR (95% CI): 0.81 (0.46, 1.42)]. There was an indication that higher urinary sucrose levels were associated with increased incidence of diabetes in the continuous analysis [HR (95% CI) per 100 µM: 1.14 (0.95, 1.36)] and comparing fourth vs. first quartile [HR (95% CI): 1.36 (0.77, 2.41)]. After including BMI or WC as covariates into the multivariable models the associations were attenuated (Table 2). The smoothing spline indicated that the hazard ratio of incident diabetes was increasing with higher urinary sucrose levels (Fig. 3).

**Table 2:** Hazard ratios with 95% confidence intervals for the association between urinary and dietary sucrose and diabetes incidence (*n* = 2996).

			Model 1^a^	Model 2^b^	Model 2 + BMI	Model 2 + WC
			HR (95% CI)	HR (95% CI)	HR (95% CI)	HR (95% CI)
*Sucrose intake - FFQ*	*n*	cases				
Q1 (3.0–32.3 g)	*n* = 749	*n* = 26	1.00	1.00	1.00	1.00
Q2 (32.3–44.0 g)	*n* = 749	*n* = 28	1.07 (0.62, 1.82)	1.06 (0.61, 1.86)	1.15 (0.66, 2.01)	1.18 (0.68, 2.07)
Q3 (44.0–57.1 g)	*n* = 749	*n* = 23	0.85 (0.49, 1.50)	0.86 (0.45, 1.62)	1.00 (0.53, 1.89)	1.08 (0.57, 2.04)
Q4 (57.2–76.3 g)	*n* = 749	*n* = 20	0.72 (0.40, 1.30)	0.67 (0.30, 1.53)	0.82 (0.36, 1.84)	0.88 (0.39, 1.99)
pTrend			0.19	0.27	0.53	0.66
Per 50 g/d	*n* = 2996	*n* = 97	0.82 (0.56, 1.20)	0.81 (0.46, 1.42)	1.01 (0.58, 1.76)	1.03 (0.59, 1.81)
*Sucrose intake - 7DD*	*n*	cases				
Q1 (0.5–28.1 g)	*n* = 749	*n* = 26	1.00	1.00	1.00	1.00
Q2 (28.1–38.4 g)	*n* = 749	*n* = 24	0.87 (0.50, 1.52)	0.92 (0.52, 1.63)	0.99 (0.55, 1.76)	1.03 (0.58, 1.85)
Q3 (38.5–49.5 g)	*n* = 749	*n* = 26	0.95 (0.55, 1.64)	1.00 (0.55, 1.80)	1.21 (0.67, 2.21)	1.29 (0.71, 2.35)
Q4 (49.5–65.9 g)	*n* = 749	*n* = 21	0.74 (0.41, 1.33)	0.75 (0.37, 1.51)	0.96 (0.47, 1.94)	0.95 (0.47, 1.91)
pTrend			0.35	0.46	1.00	0.95
Per 50 g/d	*n* = 2996	*n* = 97	0.67 (0.49, 0.92)	0.63 (0.43, 0.91)	0.76 (0.52, 1.10)	0.79 (0.55, 1.14)
*Urinary sucrose*	*n*	cases				
Q1 (5.0–13.7 µM)	*n* = 749	*n* = 21	1.00	1.00	1.00	1.00
Q2 (13.7–32.6 µM)	*n* = 749	*n* = 23	1.08 (0.60, 2.00)	1.11 (0.61, 2.02)	1.07 (0.59, 1.94)	1.12 (0.62, 2.04)
Q3 (32.6–73.0 µM)	*n* = 749	*n* = 24	1.15 (0.64, 2.06)	1.18 (0.65, 2.12)	1.09 (0.61, 1.96)	1.11 (0.62, 2.00)
Q4 (73.3–499.9 µM)	*n* = 749	*n* = 29	1.37 (0.77, 2.41)	1.36 (0.77, 2.41)	1.21 (0.68, 2.14)	1.21 (0.68, 2.14)
pTrend			0.25	0.30	0.51	0.58
Per 100 µM	*n* = 2996	*n* = 97	1.14 (0.95, 1.37)	1.14 (0.95, 1.36)	1.08 (0.90, 1.30)	1.07 (0.90, 1.29)

^a^Adjusted for age and sex.

^b^Adjusted for age, sex, total energy, education level, smoking status, physical activity level, and family history of diabetes + height (for WC).

In the sensitivity analysis with imputed values outside the detection limits, sucrose assessed with FFQ and 7DD (per 50 g/d) was associated with a 34% [HR (95% CI): 0.66 (0.44, 0.98)] and 29% [HR (95% CI): 0.71 (0.52, 0.96)] lower hazard of developing diabetes in the adjusted model (model 2), respectively (Supplementary Table 2). Urinary sucrose per 100 µM was not associated with diabetes incidence [HR (95% CI): 1.00 (0.92, 1.08)]. However, comparing the fourth vs. first quartile of urinary sucrose pointed to an increase in T2D incidence [HR (95% CI): 1.38 (0.79, 2.42)].

Regarding the criteria for mediation analysis, urinary sucrose showed a positive association with both, BMI and WC (Supplementary Table 3). BMI and WC were also associated with diabetes incidence (Supplementary Table 4). Findings from the mediation analysis, using BMI as the mediator, pointed to an increase in the hazard of incident diabetes for the TIE [HR (95% CI): 1.02 (1.00, 1.04)] and the PDE [HR (95% CI): 1.11 (0.92, 1.35)] for urinary sucrose per 100 µM. The PM of BMI in this relation was 16%. The findings were similar, when WC was used as a mediator (WC: PM: 22%). In sensitivity analysis using BMI and WC from 2HC as mediators, findings on PDE, TIE, and total effect were comparable [BMI: TIE (95% CI): 1.02 (0.99, 1.06), PM: 10%; WC: TIE (95% CI): 1.03 (1.00, 1.07), PM: 12%] (Table 3). Plotting the causal effects estimated as a function of time showed that the HRs for diabetes incidence did not vary over the follow-up period for the PDE, TIE, and total effect (Supplementary Fig. 4).

**Table 3:** Mediation analysis of hazard ratios for BMI or WC from baseline (BL—*n* = 2996) and second health check (2HC—*n* = 1728) on the association between urinary sucrose (per 100 µM) and diabetes incidence.

	Mediator: BMI (BL)	Mediator: WC (BL)
	HR (95% CI)	HR (95% CI)
Urinary sucrose per 100 µM		
Pure direct effect	1.11 (0.92, 1.35)	1.12 (0.91, 1.39)
Total indirect effect	1.02 (1.00, 1.04)	1.03 (1.01, 1.06)
Total effect	1.13 (0.94, 1.37)	1.16 (0.94, 1.42)
Proportion mediated	16%	22%

	Mediator: BMI (2HC)	Mediator: WC (2HC)
	HR (95% CI)	HR (95% CI)
Urinary sucrose per 100 µM		
Pure direct effect	1.25 (0.96, 1.63)	1.31 (0.95, 1.80)
Total indirect effect	1.02 (0.99, 1.06)	1.03 (1.00, 1.07)
Total effect	1.28 (0.99, 1.65)	1.35 (0.99, 1.83)
Proportion mediated	10%	12%

All models are adjusted for age, sex, total energy, education level, smoking status, physical activity level, and family history of diabetes + height (for WC).

## Discussion

The findings of our study indicated inverse associations for dietary sucrose assessed via FFQ and 7DD with diabetes incidence, but a tendency to a positive association for urinary sucrose regarding incident diabetes. Results from the mediation analysis suggested that the total association between urinary sucrose and diabetes incidence might be explained by ~16% or 22% by the indirect pathway via BMI or WC. However, the effect size of the TIE is small and the PDE is imprecisely estimated. Nevertheless, it is possible that direct or other mechanisms play a role and influence this association.

Our results reflect the present body of research, indicating that the evidence for associations between dietary sugar intake and incident diabetes is inconsistent [4]. Whereas there is a high certainty of the evidence for a positive association between sugar-sweetened beverages and diabetes incidence [2–4], the evidence regarding dietary sugar intake as a nutrient remains inconclusive and findings are controversial [4, 15]. Similar to the findings of a systematic review and meta-analysis, in which sucrose intake was inversely associated with the incidence of T2D [15], we also found an inverse association for dietary sucrose assessed with 7DD, and an imprecisely estimated inverse association for dietary sucrose assessed with FFQ regarding the incidence of diabetes. Moreover, some studies showed that the inverse association between dietary sugar intake and diabetes incidence disappeared after adjusting for BMI [6, 8, 9]. In this study, the estimated associations were also attenuated after including BMI and WC in the adjusted Cox proportional hazard models.

The results on urinary sucrose point to a positive association regarding diabetes incidence. This observation is in line with existing studies using urinary biomarkers in adults. In feeding studies, conducted in the UK [24, 33] and USA [34], 24-hour urinary sucrose and fructose (24uSF) were used to develop a predictive objective biomarker for dietary sugar intake. A validation of the biomarker, considering different levels of specific biases, was performed to assess “unbiased” sugar intake [35]. In the Women’s Health Initiative, dietary sugar assessed by self-reports was associated with a decreased risk of T2D, but after using the calibrated intake using equations from the biomarker study, findings were attenuated towards the null [36]. Beyond that, studies investigating sugar intake and risk of obesity also found a positive association using 24uSF [37], and urinary sucrose from spot urine [18, 19], whereas self-reported dietary sugar intake was not associated with obesity. Furthermore, findings of a Swedish study showed positive associations for overnight urinary sucrose and fructose with BMI, WC, blood pressure, and plasma fasting glucose in women. However, interestingly, the urinary biomarker was inversely associated with BMI and WC in men. For self-reported dietary sugar intake, no associations were observed in this study for most of the outcomes [38]. Using BMI and WC as mediators, the findings of our study indicate that overweight and obesity may partly explain the association between sucrose intake and diabetes incidence. Here, the mediating effect of BMI was weaker compared to the findings in our ecological mediation analysis based on aggregated data [BMI: PM (95% CI): 66% (34%, 100%)] [21]. Consequently, there is an indication that direct mechanisms next to overweight and obesity might have an impact on the association between sugar intake and the development of diabetes.

The different findings regarding urinary sugar biomarkers and self-reported dietary sugar intake may be explained due to reporting bias. Dietary data is usually based on self-reports of participants using questionnaires, which is prone to bias, and especially overweight individuals tend to underreport their true intake [16, 17]. In studies that investigated differences between 24uSF as an objective urinary biomarker and self-reported dietary sugar intake, biomarker predicted sugar intake was higher than self-reported dietary sugar intake, indicating measurement errors and underreporting due to self-reports [39, 40]. Feeding studies were conducted to investigate 24uSF as a potential predictive and objective biomarker [24, 33, 34], and one of those studies reported a high correlation between 30-days mean 24uSF and 30-days mean dietary sugar intake [Spearman correlation coefficient (95% CI): 0.84 (0.54, 0.95)] [24]. Consequently, a calibration study was carried out in the Observing Protein and Energy Nutrition (OPEN) study, in order to find a reliable biomarker for objective dietary sugar assessment [35]. Yet, 24uSF was used in several cohorts [35–37, 39, 40], whereas, other studies were restricted to urinary sucrose or fructose from spot urine samples [18, 19, 38]. However, urinary biomarkers from spot urine sample, which was also used in our study, were not proven as objective biomarkers in calibration studies yet.

The present body of research implies that a high dietary sugar intake may lead to overweight and obesity due to an overload of calories [41], and consequently, the indirect pathway via overweight and obesity is the causal risk factor for incident T2D [42]. However, other direct sugar-related pathophysiologic mechanisms might have an impact on the association between dietary sugar intake and T2D. For instance, a high dietary sugar intake, and especially fructose intake, has been associated with increased liver fat content and non-alcoholic fatty liver disease [43, 44], which are causal risk factors for insulin resistance [45]. Beyond that, a diet high in added sugars can lead to a higher glycaemic and insulinemic response due to a higher glycaemic load [46], which is associated with an increased risk of T2D [47, 48].

Our study has several strengths and limitations. This was the first study that investigated the indirect impact and mediation effect of BMI or WC on the association between dietary sugar intake and incidence of diabetes in a prospective approach using individual data. Furthermore, different assessment methods of sucrose were available and thus, results of dietary sucrose assessed with FFQ and 7DD could be compared to urinary sucrose. However, our study had some limitations as well. First, only 97 participants developed T2D during follow-up. Due to this small number, 95% CIs were wide and thus, findings were imprecisely estimated. Consequently, these findings should be interpreted with caution. Second, a large number of participants in our study population died during the follow-up period (*n* = 1358). As we considered death as a censoring event for the time to incident diagnosis, we assume that time to death and time to diabetes diagnosis are independent. However, this assumption might not be fulfilled, and thus, competing risk cannot be ruled out. Third, the urinary sucrose levels of 42.5% of the participants were below and 0.9% above the detection level of 5–500 µM (*n* = 2292). Recent studies used other methods with a lower detection limit to assess urinary sucrose, and thus, excluded a lower percentage of their participants (~4–10%) [34, 38]. We, therefore, conducted a sensitivity analysis and imputed sucrose values outside the detection limits. However, after including these imputed values the association between urinary sucrose and incidence of diabetes was u-shaped, and thus, we cannot be sure if the measured values below the detection limit represent the true values. Fourth, urinary sucrose was only measured once and assessed via spot urine samples. Urinary sucrose from spot urine was also considered in other studies [18, 19, 38], however, no validation studies have been conducted so far, that investigated whether spot urine has the potential to predict usual sucrose intake.

## Conclusions

Our findings showed that sucrose measured as objective urinary biomarker points to an increased incidence of diabetes. BMI and WC may partly mediate this association; however, it is possible that other mechanisms also play a role. More large-scale epidemiological studies considering objective sucrose biomarkers are warranted to obtain more precise results.

### Supplementary information

Supplemental material for publication

## Data Availability

The data set that supports the findings of this study cannot be made available by the authors, since they have no permission to share the data. EPIC-Norfolk researchers will make the data set available under a Data Transfer Agreement to any bona fide researcher who wishes to obtain the data set in order to undertake a replication analysis. The contact for data requests is: epic-norfolk@mrc-epid.cam.ac.uk.
