# Supplementary material for: The mediating role of obesity on the prospective association between urinary sucrose and diabetes incidence in a sub-cohort of the EPIC-Norfolk
Source: Nutr Diabetes. 2023 Sep 2;13:14. doi: 10.1038/s41387-023-00243-5 (PMC10475125; doi:10.1038/s41387-023-00243-5)
Supplement: Supplementary file 1 — Supplemental material for publication [file 41387_2023_243_MOESM1_ESM.docx]

Online supplemental material

The mediating role of BMI on the prospective association between dietary and urinary sucrose with diabetes incidence in a sub-cohort of the EPIC-Norfolk

Alexander Lang^1^, Oliver Kuss^1,2,3^, Tim Filla^4^, Gunter Kuhnle^5^, Sabrina Schlesinger^1,3^

^1^ Institute for Biometrics and Epidemiology, German Diabetes Center, Leibniz Institute for Diabetes Research at Heinrich Heine University Düsseldorf, Auf'm Hennekamp 65, D-40225 Düsseldorf, Germany

^2^ Centre for Health and Society, Medical Faculty and University Hospital Düsseldorf, Heinrich-Heine-University Düsseldorf, Germany Institute for Biometrics and Bioinformatics, University Hospital

^3^ German Center for Diabetes Research, Partner Düsseldorf, München-Neuherberg, Germany

^4^ Department of Rheumatology, Medical Faculty, Heinrich Heine University Düsseldorf, Düsseldorf, Germany

^5^ Department of Food & Nutritional Sciences, University of Reading, Reading RG6 6DZ, United Kingdom

Corresponding author: Alexander Lang, German Diabetes Center, Institute for Biometrics and Epidemiology, Research Group Systematic Reviews, Leibniz Center for Diabetes Research at Heinrich Heine University Düsseldorf, Auf’m Hennekamp 65, 40225 Düsseldorf, Germany, phone: +49-(0)-211-33-82-355, e-mail: alexander.lang@ddz.de

Supplement Table 1: Sensitivity analysis: Baseline characteristics of participants in a sub-group of EPIC-Norfolk after multiple imputation (n=5 288)

|  | **Group 1**  **(0.0 - 5.0 µM)** | **Group 2**  **(>5.0 - 13.8 µM)** | **Group 3**  **(>13.8 - 33.3 µM)** | **Group 4**  **(>33.3 - 76.3 µM)** | **Group 5**  **(>76.3 - 1000.0 µM)** | **Total**  **(0.0 - 1000.0 µM)** |
| --- | --- | --- | --- | --- | --- | --- |
| n | 2246 | 760 | 760 | 761 | 761 | 5288 |
| n incident diabetes | 65 | 21 | 24 | 24 | 31 | 165 |
| Age [years] | 59.3 ± 9.0 | 59.4 ± 9.3 | 60.4 ± 9.4 | 60.3 ± 9.4 | 62.4 ± 9.5 | 60.1 ± 9.3 |
| Sex [m/f] | 36.0% / 64.0% | 39.3% / 60.7% | 47.2% / 52.8% | 48.9% / 51.1% | 54.8% / 45.2% | 42.6% / 57.4% |
| Body mass index [kg/m²] | 26.1 ± 3.8 | 26.0 ± 3.8 | 26.1 ± 3.7 | 26.4 ± 3.8 | 26.6 ± 3.9 | 26.2 ± 3.8 |
| <25 kg/m² | 41.3% | 44.2% | 40.3% | 37.3% | 38.8% | 40.6% |
| 25-<30 kg/m² | 44.9% | 43.7% | 46.6% | 46.4% | 43.6% | 45.0% |
| ≥30 kg/m² | 13.8% | 12.1% | 13.2% | 16.3% | 17.6% | 14.4% |
| Waist circumference [cm] | 85.9 ± 11.8 | 86.6 ± 12.2 | 87.8 ± 11.9 | 89.2 ± 11.8 | 90.4 ± 12.4 | 87.4 ± 12.1 |
| <80cm (f), <94 cm (m) | 50.7% | 49.3% | 46.7% | 43.9% | 43.6% | 47.9% |
| 80-<88 cm (f), 94-<102cm (m) | 26.8% | 29.3% | 29.2% | 30.2% | 28.3% | 28.2% |
| ≥88 cm (f), ≥102 cm (m) | 22.4% | 21.3% | 24.1% | 25.9% | 28.1% | 23.8% |
| Height | 165 ± 8.8 | 166 ± 9.0 | 167 ± 9.0 | 168 ± 9.4 | 168 ± 9.4 | 166 ± 9.1 |
| Total energy (FFQ) [kcal/day] | 2007 ± 552 | 2012 ± 524 | 2032 ± 553 | 2085 ± 584 | 2125 ± 596 | 2040 ± 561 |
| Total energy (7DD) [kcal/day] | 1883 ± 500 | 1924 ± 480 | 1956 ± 515 | 1983 ± 534 | 1993 ± 538 | 1930 ± 512 |
| Education level |  |  |  |  |  |  |
| none | 39.8% | 37.2% | 36.6% | 39.6% | 45.9% | 39.8% |
| O-level | 9.6% | 10.5% | 10.8% | 9.7% | 9.1% | 9.8% |
| A-level | 38.3% | 39.6% | 38.6% | 38.4% | 36.5% | 38.3% |
| degree | 12.3% | 12.6% | 14.1% | 12.4% | 8.5% | 12.1% |
| Smoking status |  |  |  |  |  |  |
| current | 9.3% | 8.2% | 6.7% | 9.7% | 8.8% | 8.8% |
| former | 40.0% | 42.8% | 46.8% | 45.1% | 48.6% | 43.4% |
| never | 50.6% | 49.1% | 46.4% | 45.2% | 42.6% | 47.9% |
| Physical activity level |  |  |  |  |  |  |
| inactive | 25.5% | 26.4% | 28.8% | 27.6% | 34.0% | 27.6% |
| moderately inactive | 30.7% | 30.3% | 30.0% | 28.3% | 27.7% | 29.7% |
| moderately active | 24.1% | 23.6% | 24.2% | 23.9% | 21.0% | 23.6% |
| active | 19.8% | 19.7% | 17.0% | 20.2% | 17.2% | 19.1% |
| Family history of diabetes |  |  |  |  |  |  |
| yes | 10.9% | 13.6% | 10.5% | 11.6% | 11.3% | 11.4% |
| no | 89.1% | 86.4% | 89.5% | 88.4% | 88.7% | 88.6% |

Supplement Table 2: Sensitivity analysis: Hazard ratios with 95% confidence intervals after imputation (n=5 288)

* adjusted for age and sex

** adjusted for age, sex, total energy, education level, smoking status, physical activity level and family history of diabetes + height (for WC)

|  |  |  | Model 1* | Model 2** | Model 2 + BMI | Model 2 + WC |
| --- | --- | --- | --- | --- | --- | --- |
|  |  |  |  |  |  |  |
|  |  |  | HR (95% CI) | HR (95% CI) | HR (95% CI) | HR (95% CI) |
|  | persons | cases | *Sucrose intake - FFQ* | | | |
| Q1 (3.0 - 32.3 g) | n = 1058 | n = 44 | 1.00 | 1.00 | 1.00 | 1.00 |
| Q2 (32.3 - 44.0 g) | n = 1057 | n = 35 | 0.75 (0.48, 1.17) | 0.74 (0.47, 1.17) | 0.81 (0.51, 1.28) | 0.84 (0.53, 1.33) |
| Q3 (44.0 - 57.1 g) | n = 1058 | n = 24 | 0.51 (0.31, 0.84) | 0.50 (0.29, 0.85) | 0.60 (0.35, 1.03) | 0.63 (0.37, 1.07) |
| Q4 (57.2 - 76.3 g) | n = 1057 | n = 32 | 0.65 (0.41, 1.03) | 0.63 (0.37, 1.09) | 0.76 (0.44, 1.30) | 0.79 (0.46, 1.36) |
| Q5 (76.4 - 280.6 g) | n = 1058 | n = 30 | 0.56 (0.35, 0.90) | 0.51 (0.27, 0.99) | 0.67 (0.35, 1.28) | 0.70 (0.36, 1.35) |
| pTrend |  |  | 0.03 | 0.07 | 0.30 | 0.36 |
| Per 50 g/d | n = 5288 | n = 165 | 0.70 (0.53, 0.94) | 0.66 (0.44, 0.98) | 0.78 (0.52, 1.17) | 0.80 (0.53, 1.20) |
|  | persons | cases | *Sucrose intake - 7DD* | | | |
| Q1 (0.5 - 28.1 g) | n = 1058 | n = 42 | 1.00 | 1.00 | 1.00 | 1.00 |
| Q2 (28.1 - 38.4 g) | n = 1057 | n = 28 | 0.65 (0.40, 1.05) | 0.69 (0.42, 1.13) | 0.75 (0.46, 1.23) | 0.80 (0.49, 1.30) |
| Q3 (38.5 - 49.5 g) | n = 1058 | n = 33 | 0.74 (0.47, 1.17) | 0.83 (0.51, 1.35) | 0.96 (0.59, 1.56) | 0.99 (0.61, 1.62) |
| Q4 (49.5 - 65.9 g) | n = 1057 | n = 27 | 0.58 (0.36, 0.94) | 0.63 (0.37, 1.07) | 0.80 (0.47, 1.37) | 0.84 (0.49, 1.43) |
| Q5 (65.9 - 199.6 g) | n = 1058 | n = 35 | 0.71 (0.44, 1.12) | 0.73 (0.42, 1.28) | 0.97 (0.55, 1.70) | 0.96 (0.55, 1.67) |
| pTrend |  |  | 0.18 | 0.34 | 0.93 | 1.00 |
| Per 50 g/d | n = 5288 | n = 165 | 0.72 (0.56, 0.92) | 0.71 (0.52, 0.96) | 0.83 (0.60, 1.13) | 0.86 (0.63, 1.16) |
|  | persons | cases | *Urinary sucrose* | | | |
| Q1 (0.0 - 5.0 µMol) | n = 2246 | n = 65 | 1.06 (0.65, 1.74) | 1.12 (0.68, 1.83) | 1.11 (0.68, 1.83) | 1.14 (0.70, 1.87) |
| Q2 (5.0 - 13.8 µMol) | n = 761 | n = 21 | 1.00 | 1.00 | 1.00 | 1.00 |
| Q3 (13.9 - 33.3 µMol) | n = 760 | n = 24 | 1.09 (0.61, 1.97) | 1.14 (0.63, 2.05) | 1.12 (0.62, 2.01) | 1.17 (0.65, 2.11) |
| Q4 (33.3 - 76.3 µMol) | n = 760 | n = 24 | 1.11 (0.62, 1.99) | 1.15 (0.64, 2.06) | 1.07 (0.60, 1.93) | 1.08 (0.60, 1.94) |
| Q5 (76.3 - 505.0 µMol) | n = 761 | n = 31 | 1.37 (0.78, 2.39) | 1.38 (0.79, 2.42) | 1.25 (0.71, 2.19) | 1.25 (0.71, 2.18) |
| pTrend |  |  | 0.08 | 0.11 | 0.28 | 0.34 |
| Per 100 µM | n = 5288 | n = 165 | 1.00 (0.92, 1.08) | 1.00 (0.92, 1.08) | 0.99 (0.92, 1.07) | 0.99 (0.92, 1.07) |

Supplement Table 3: Results of linear and logistic regression for the association between urinary and dietary sucrose and BMI or WC

All models are adjusted for age, sex, total energy, education level, smoking status, physical activity level and family history of diabetes

* for logistic regression analysis: BMI ≥30 kg/m²

** further adjustment for height; for logistic regression analysis: WC ≥ 88 cm (f) and ≥ 102 cm (m)

|  | BMI* | | WC** | |
| --- | --- | --- | --- | --- |
|  | β-estimate (95% CI) | OR (95% CI) | β-estimate (95% CI) | OR (95% CI) |
| Urinary sucrose per 100 µM | 0.21 (0.09, 0.33) | 1.20 (1.09, 1.31) | 0.51 (0.19, 0.84) | 1.12 (1.04, 1.21) |

Supplement Table 4: Results of Cox regression for the association between BMI or WC and diabetes incidence

All models are adjusted for age, sex, total energy, education level, smoking status, physical activity level and family history of diabetes + height (for WC)

|  | Diabetes incidence |
| --- | --- |
|  | HR (95% CI) |
| BMI [per kg/m²] | 1.16 (1.12, 1.21) |
| WC [per cm] | 1.07 (1.06, 1.09) |


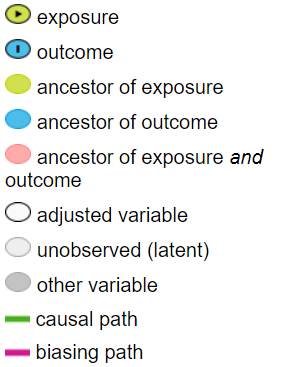

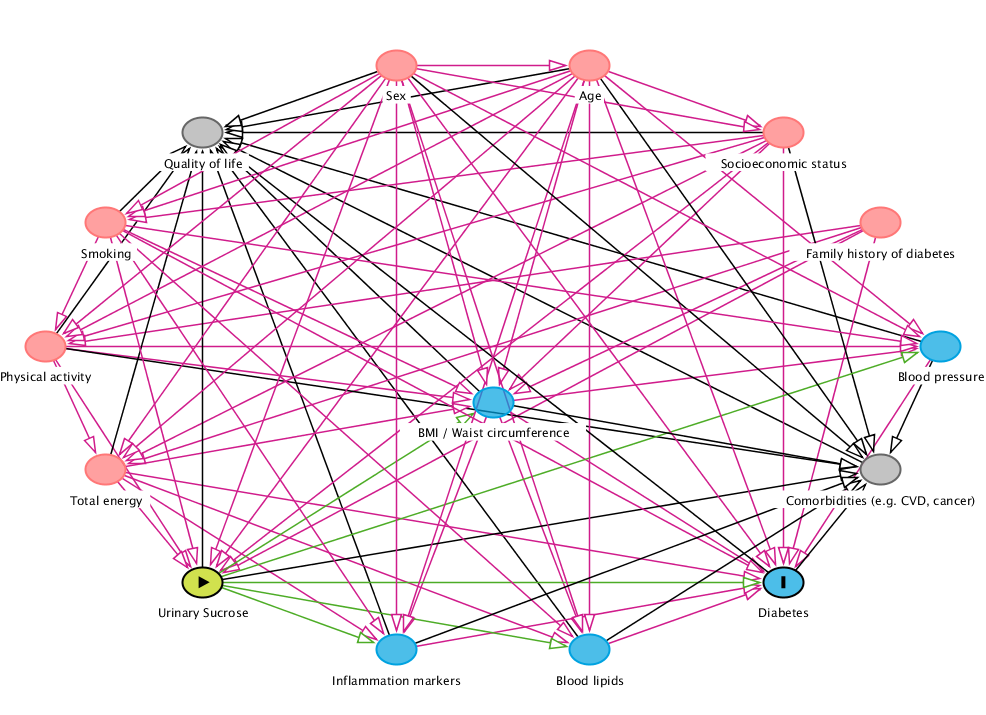

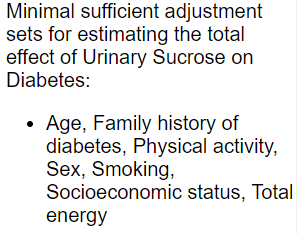

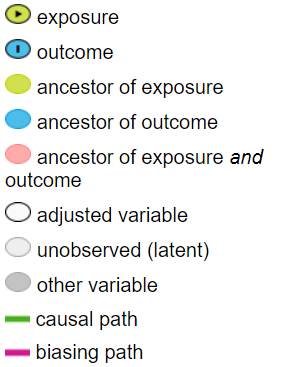

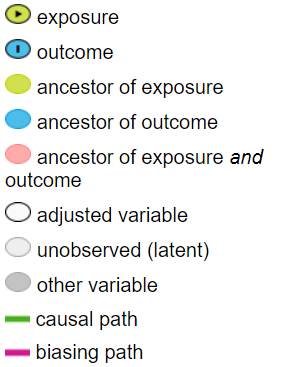


Supplement Figure 1: Directed acyclic graphs (DAGs) for the investigated association between the exposure (urinary sucrose), and the outcome (diabetes) with the mediator (BMI / Waist circumference)


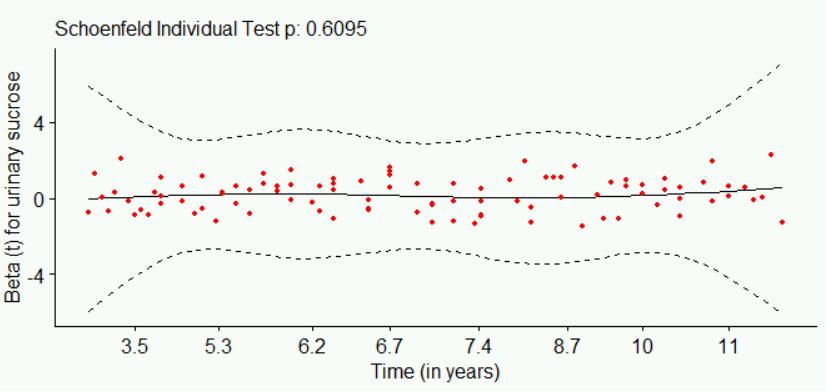


**A**


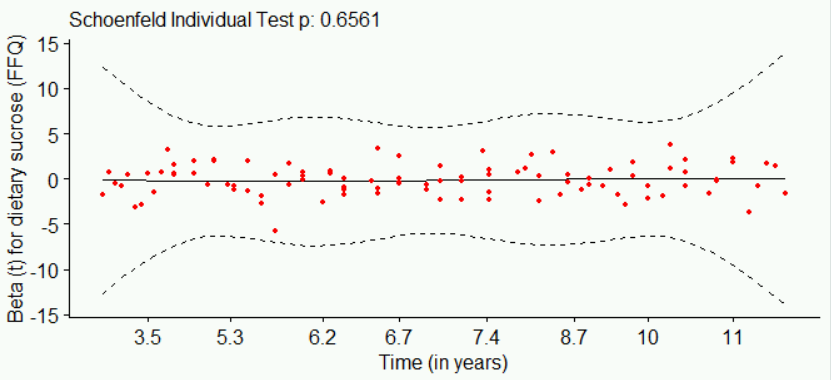


**B**


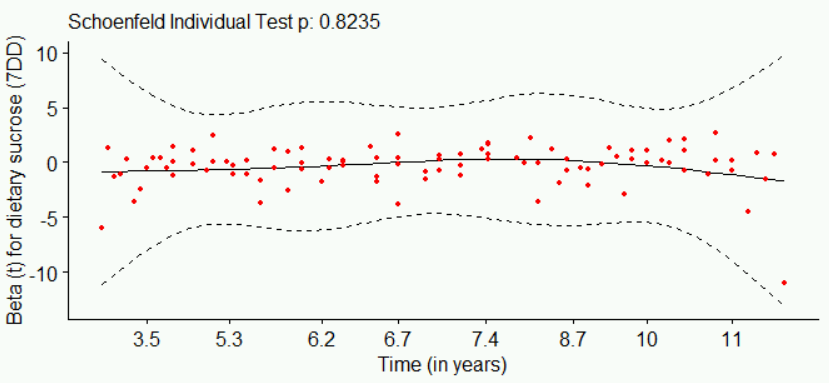


**C**

Supplement Figure 2: Schoenfeld residuals of urinary sucrose (A) and dietary sucrose assessed with FFQ (B) and 7DD (C) with the confounding variables


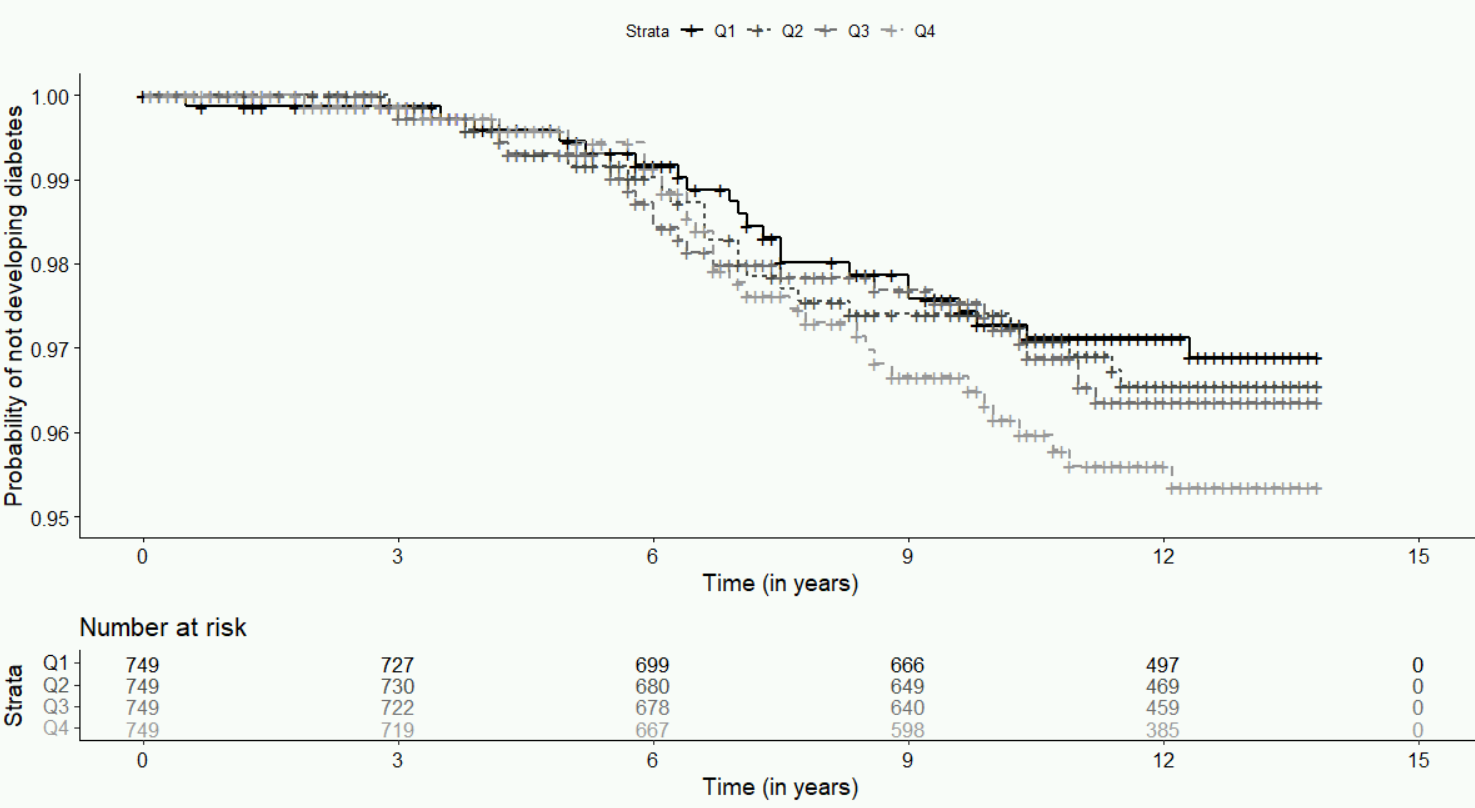

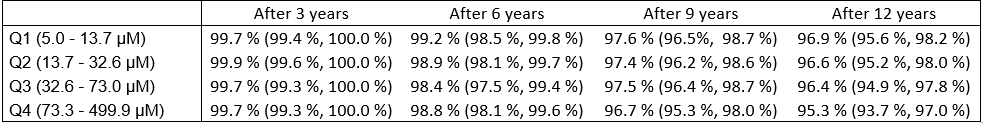


Supplement Figure 3: Survival analysis of the quartiles of urinary sucrose and probability of not developing diabetes over follow-up time (n=2 996)


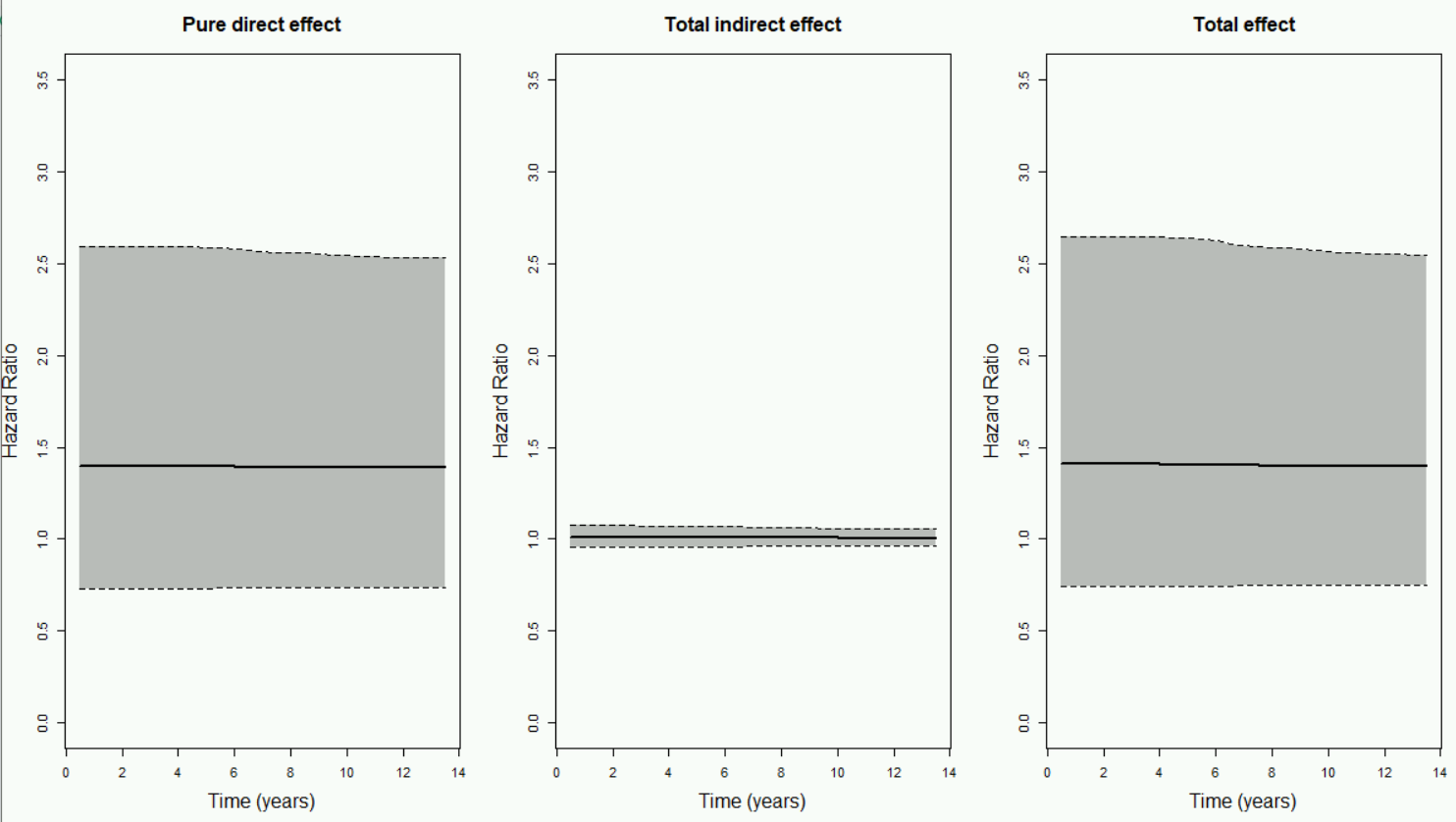


Supplement Figure 4: Causal effects of the pure direct effect, total indirect effect and total effect estimated as a function of time to event (years) comparing highest vs lowest quartile (Q4 vs. Q1) of urinary sucrose, with BMI as mediator and diabetes incidence
